# Adolescent Nonsuicidal Self-Injury and Suicidality: A Latent Class Analysis and Associations with Clinical Characteristics in an At-Risk Cohort

**DOI:** 10.1007/s10964-023-01922-3

**Published:** 2023-12-19

**Authors:** N. G. M. de Neve-Enthoven, A. P. Ringoot, J. Jongerling, N. Boersma, L. M. Berges, D. Meijnckens, W. J. G. Hoogendijk, N. H. Grootendorst-van Mil

**Affiliations:** 1 Department of Psychiatry, Erasmus MC University Medical Center, Rotterdam, the Netherlands; 2 Department of Psychology and Educational Sciences, Open University of the Netherlands, Heerlen, the Netherlands; 3 Department of Psychology, Educational and Child Studies, Erasmus University Rotterdam, Rotterdam, the Netherlands; 4 Tilburg School of Social and Behavioral Sciences, Department of Methodology, Tilburg University, Tilburg, the Netherlands; 5 Clinical Psychologist at Yulius, Dordrecht, the Netherlands; 6 MIND Platform, Utrecht, the Netherlands; 7 Stichting Zelfbeschadiging (Self-harm Foundation), Utrecht, the Netherlands; 8 Epidemiological and Social Psychiatric Research Institute (ESPRi), Erasmus MC University Medical Center, Rotterdam, the Netherlands

**Keywords:** Nonsuicidal self-injury, Suicidality, Adolescents, Latent class analysis, Population-based

## Abstract

Nonsuicidal self-injury (NSSI) is frequently encountered in adolescents, but its predictive value for suicidality or other clinical characteristics is challenging due to its heterogeneous nature. This study used latent class analysis to identify subgroups of NSSI and compared these on sociodemographic characteristics, adverse outcomes and protective factors. The study included 966 high-risk adolescents, *M*age 14.9 y, *SD* 0.9 y, 51.8% female. Four classes emerged: (1) “Low NSSI–Low suicidality”, (2) “Moderate NSSI-Low suicidality”, (3) “Moderate NSSI-High suicidality”, and (4) “High NSSI-High suicidality”. Girls predominated in the high suicidality classes. Generally, Class 4 had the poorest outcomes: more internalizing and externalizing problems, less social support from friends and families and worst self-esteem. These findings emphasize the need for interventions tailored to specific phenotypes of adolescents engaging in NSSI.

## Introduction

Adolescence is a critical period marked by various developmental challenges, including identity formation, emotional regulation, changes in interpersonal relationships, and an increasing vulnerability to emotional and behavioral problems associated with poor emotion regulation (Ahmed et al., 2015). Amidst these challenges, a significant number of adolescents turn to nonsuicidal self-injury (NSSI), which is defined as the intentional destruction of one’s body tissue without suicidal intent and for purposes not socially sanctioned, e.g., cutting or burning (Nock, 2010). NSSI can be used as a coping mechanism to manage emotional distress, regulate overwhelming emotions, or express inner turmoil (Klonsky, 2007). It is a relatively common, yet complex and alarming phenomenon that has garnered significant attention from researchers and clinicians alike because of its association with emotional and psychiatric distress (Klonsky et al., 2014). Multiple longitudinal studies have found that self-injurious behavior in adolescence predicts adverse outcomes in young adulthood, such as substance use, mental health problems, financial and social problems, and future self-harm, be it nonsuicidal or suicidal (Borschmann et al., 2017; Daukantaitė et al., 2021; Mars et al., 2014; Moran et al., 2012). This highlights the urgency for a comprehensive understanding of its underlying factors to be able to develop effective interventions. Despite the progress made in understanding NSSI, several gaps in the existing literature on adolescent NSSI remain because of its heterogeneous nature. Findings on specific constructs related to adolescent NSSI in general cannot be translated to all adolescents performing this behavior. The present study focuses on differences on sociodemographic characteristics and adverse outcomes, as well as protective factors in subgroups of adolescents performing NSSI.

Prevalence rates of NSSI in adolescents and young adults found in previous studies are highly variable. For example, rates of 17.2% in adolescents and 13.4% in young adults from nonclinical samples (Swannell et al., 2014), while prevalence rates of 40–80% have been reported in adolescent inpatient samples (Klonsky & Muehlenkamp, 2007). Studying possible associations between NSSI and suicidality (comprising thoughts, plans, and behaviors related to suicide) is complex and multifaceted. Several studies have highlighted important distinctions between NSSI and suicidality, such as the intent behind the behavior, the nature of the self-injurious acts, and the specific psychological processes involved (Dhingra et al., 2016). In contrast, other studies explored the overlap and potential pathways connecting NSSI and suicidality. Findings from numerous cross-sectional and longitudinal studies have provided valuable insights into the co-occurrence of NSSI and suicidality, highlighting the increased risk of suicidal thoughts and suicide attempts among individuals who engage in NSSI (Kiekens et al., 2018; Ribeiro et al., 2016; Victor & Klonsky, 2014). A history of NSSI is a robust predictor for suicidality, beyond the effects of other risk factors, with a younger age of NSSI onset being a risk factor for more severe NSSI and suicidal behavior (Grandclerc et al., 2016; Muehlenkamp et al., 2019). Nevertheless, it should be noted that according to the *antisuicide model*, for some individuals, endorsing NSSI serves as an active coping mechanism to avoid suicide (Suyemoto, 1998).

Several studies have employed latent class analysis (LCA) to investigate patterns and subgroups of NSSI in various populations. LCA is a statistical technique that identifies distinct and homogeneous classes or groups of individuals based on patterns of responses to a set of variables (Asparouhov & Muthén, 2014). In LCA, a person-centered approach is used, which means that the primary interest is on relationships among individuals rather than relationships among variables (Hamza & Willoughby, 2013). By applying LCA, researchers attempt to uncover meaningful typologies of NSSI behaviors, which can inform clinical understanding, intervention strategies, and prevention efforts. Findings from previous studies using LCA to classify NSSI have identified distinct classes of persons endorsing NSSI. Class indicators in these models were predominantly NSSI method and NSSI frequency (Case et al. (2020); Chen & Chun, 2019; Peterson et al., 2019; Reinhardt et al., 2021; Reinhardt et al., 2022; Somer et al., 2015), although some studies added circumstantial characteristics, such as the urgency to act and the feeling of pain during self-harm, or motivational background as additional indicators (Klonsky & Olino, 2008; Whitlock et al., 2008).

Few studies performing LCA have combined NSSI *and* suicidality as class indicators. In a study in an adult acute inpatient hospital setting both a low- and a high-risk group for self-injurious thoughts and behaviors were identified (Dhingra et al., 2015). A study in a sample of university students endorsing NSSI identified three classes: a low-risk group characterized by minimal NSSI and low suicidality, a moderate-risk group with NSSI and suicidal ideation, and a high-risk group exhibiting suicidal behavior (Dhingra et al., 2016). Researchers identified three classes in a sample of first-year undergraduates: an infrequent NSSI/not high risk for suicidal behavior group, a frequent NSSI/not high risk for suicidal behavior group, and a frequent NSSI/high risk for suicidal behavior group (Hamza & Willoughby, 2013). Results from an LCA in a college sample supported a 3-class solution as well, with students classified as being likely to have no history of any self-injurious thoughts and behaviors, a history of all self-injurious thoughts and behaviors measured, or a history of suicidal ideation, plan, and nonsuicidal self-injurious thoughts and behaviors, but not suicide attempt (Marraccini et al. (2021)). Notably, in all previous studies, individuals in the classes with the most frequent nonsuicidal self-injury and the highest levels of suicidality had the worst clinical outcomes. They reported higher rates of co-occurring mental health conditions, such as depression, anxiety, or borderline personality disorder, and more problematic behavioral outcomes, while other classes showed lower rates of psychiatric comorbidity (Dhingra et al., 2016; Dhingra et al., 2015; Hamza & Willoughby, 2013; Marraccini et al. (2021)).

The underlying mechanisms linking NSSI and suicidality require further exploration, as well as the identification of risk and protective factors that may mitigate the risk of transitioning from NSSI to suicidal behavior. Adolescent females tend to have higher rates of NSSI (Sornberger et al., 2012) and suicide attempts (Miranda-Mendizabal et al., 2019) compared to their male counterparts. Results of studies on NSSI in adolescents belonging to ethnic minorities in Europe are scarce and have shown inconsistent results (Donath et al., 2019). Some suggest an association with socioeconomic status since economic hardship, financial instability, and related stressors can contribute to feelings of hopelessness, anxiety, and depression, which are known risk factors for self-harm. Adolescents from economically disadvantaged backgrounds tend to be at higher risk for self-harm compared to those from more affluent households (Lodebo et al., 2017). Research on the relationship between self-harm and IQ in adults has yielded mixed results. In contrast to findings in adult samples on a lower IQ being a risk factor for self-injurious behavior, a British study found that *higher* IQ was associated with an increased risk of NSSI in male and female adolescents and suicidal thoughts in males (Chang et al., 2014). Internalizing problems, which include disorders like depression and anxiety, are commonly observed alongside self-harm behaviors (Bentley et al., 2015). The relationship between self-harm and internalizing problems is often bidirectional. This means that individuals with internalizing problems are more likely to engage in self-harm, and vice versa. Although less studied, it has been found that adolescents with externalizing problems are at higher risk for engaging in self-harm as well. Aggressive behaviors may serve as a precursor or correlate of self-harm, especially in cases where individuals have difficulty regulating their emotions (Tang et al., 2013). Adequate family functioning (Diamond et al., 2022; Wang et al., 2022), ample social support (Hankin & Abela, 2011), and sufficient self-esteem (Garisch & Wilson, 2015) can act as protective factors against self-harm. These factors can help adolescents cope with stressors and seek appropriate help when needed.

## Current Study

Variations in measurement tools, inconsistent definitions, and limitations in study design have led to inconsistencies and discrepancies in previous findings. Additionally, the majority of research combining NSSI and suicidality as class indicators has focused on late adolescents, student populations, adults, or clinical samples. This limits our understanding of these phenomena in the phase of early to middle adolescence, in which both behaviors typically first emerge (Plener et al., 2015). Replication and validation of identified classes across diverse samples are necessary to ensure the generalizability of findings.

The present study is an empirical investigation of heterogeneity in NSSI and suicidality to identify possible clinical subtypes within a large, well-characterized cohort consisting of young adolescents from the general population who are at risk of the development of psychopathology (Grootendorst-van Mil et al., 2021). Through latent class analysis, the study determined how different types, frequencies, methods, and functions of NSSI cluster together and how they are associated with suicidal ideations and suicide attempts. This enabled the researchers to identify adolescents with a history of NSSI who are most at risk for suicidality. Furthermore, latent classes were compared on several sociodemographic characteristics, clinical outcomes (internalizing and externalizing problems), and protective factors (self-esteem, family functioning and social support). The results contribute to a better understanding of the risk and protective factors for NSSI and help determine which adolescents need (preventative) interventions, on which domains interventions are needed, and subsequently facilitating successful mental health care implementation.

## Method

### Participants and Procedure

The present study was embedded in the iBerry Study, a population-based longitudinal cohort study executed by the psychiatry department of the Erasmus MC University Medical Center in Rotterdam, the Netherlands. The iBerry Study examines the transition from subclinical psychiatric symptoms to psychiatric disorders by biannually assessing adolescents and a parent or primary caregiver. The study design, selection process, response rate and measurements have been described elsewhere (Grootendorst-van Mil et al., 2021). In short, in the years 2014–2015 and 2015–2016 a total of 16.736 first-year secondary school adolescents from schools located in the greater Rotterdam area completed the Strengths and Difficulties Questionnaire-Youth (SDQ-Y, (Goodman, 2001; van Widenfelt et al., 2003)) during the general medical examination executed by the community Child and Family centers. The SDQ-Y is widely used to screen for mental health in children and adolescents, with higher scores indicating more emotional and behavioral problems. For participation in the iBerry Study, the top 15% of highest-scoring adolescents and a random sample from the 85% of lowest-scoring adolescents were selected. By oversampling those adolescents with emotional and/or behavioral symptoms, the incidence of psychiatric symptoms in the cohort was increased, enabling the researchers to study developmental trajectories and causeways that underlie mental disorders.

The baseline measurements took place between September 2015 and September 2019. A series of questionnaires, cognitive measurements, interviews, and biological measures were administered at a research center for each adolescent and one or both of their parents or primary caregivers. All measurements were conducted by trained research personnel. Researchers were blinded for the adolescent’s SDQ-Y risk score. Adolescents received a small incentive for their participation. The final sample of 1022 enrolled participants (response rate: 53.9%; mean age at first visit 15.0 years) had a 2.5:1 ratio between the number of high- and lower-risk adolescents. Questionnaires on nonsuicidal self-injury and suicidality used for the present study were completed by 966 adolescents (94.5%).

The Medical Ethics Committee of the Erasmus MC University Medical Center approved the study design (MEC-015-007). All participants provided written informed consent before participation. When participants were under 16, informed consent was obtained from parents or legal guardians.

### Measures

#### Nonsuicidal self-injury

The Inventory of Statements About Self-Injury (ISAS, (Klonsky & Glenn, 2009)) is a self-report measure designed to assess NSSI behaviors performed intentionally and without suicidal intent. In Section I, the ISAS assesses the frequency of 12 methods of NSSI (e.g., cutting, scratching, burning, hitting oneself) and an open category (*Other, namely*). Adolescents were asked how often they had ever intentionally engaged in these methods. Answers to the method *Other, namely* could all be redistributed to one of the other 12 methods. They were inspected independently by a research psychologist and a clinical psychologist and in case of disagreement discussed with a psychiatrist. Two methods were excluded from further analysis since they did not qualify as NSSI: *Interference with wound healing* was excluded since the Diagnostic and Statistical Manual of Mental Disorders, 5th Edition (DSM-5, (American Psychiatric Association, 2013)) states that disturbance of wound healing or nail biting alone is insufficient to classify NSSI (American Psychiatric Association, 2013); *Swallowing toxic substances* was excluded as it was considered more indicative of suicidal behavior than NSSI. Lifetime NSSI was defined as a frequency of ≥ 1 on any of the 10 remaining methods. All methods were dummy-coded into *present* (frequency ≥1) or *absent* (frequency = 0). NSSI frequency was collapsed into four categories to create a more normalized measure of NSSI frequency (*1*, *2*–*10*, *11*–*50*, and *>50* incidents, an adaption of categorization by Hamza and Willoughby, (2013)), and the number of NSSI methods endorsed into three (*1, 2*–*3*, and *>3 behaviors*, see Whitlock et al. (2008) for a similar categorization).

Those participants endorsing lifetime NSSI were asked to fill out subsequent questions on descriptive and contextual factors surrounding the most frequently endorsed method. Items on the experience of pain during NSSI (*no, yes, sometimes*) and time between the urge to self-injure and the act (in this study collapsed into: *<1* *h, 1*–*24* *h*, > *24* *h*) were used in this study.

Section II includes 39 items that examine the motivational background for NSSI, e.g., *“When I self-harm, I am releasing emotional pressure that has been building up inside of me”*. All items can be answered on a 3-point Likert scale ranging from 0 (*not relevant at all*) to 2 (*very relevant*). The present study used the overarching Intrapersonal (i.e., affect regulation) and Interpersonal (i.e., interpersonal influence, peer-bonding) scales of the ISAS-II. The higher the scale score, the more relevant the function for the adolescent. The ISAS has been shown to have good internal consistency, test-retest reliability, and convergent and divergent validity in previous studies (Glenn & Klonsky, 2011) and good internal reliability in the present study; α = 0.82 for both subscales.

#### Suicidality

The 10-item Questions on Suicide and Self-Harm – Short Version instrument (VOZZ-screen, (Kerkhof, 2016)) was developed to identify an increased risk of suicidality in youth. All items are answered on a 5-point Likert scale with differing answering options per section of the questionnaire. The present study investigates the lifetime prevalence of suicidality and therefore used items 7 (*I have ever thought about suicide)* and 8 (*I have ever performed a suicide attempt)* to identify this construct. Both items can be answered on a Likert scale ranging from 1 *(never)* to 5 *(very often*). For data analysis, scores on both suicidal ideation and suicide attempts were dichotomized into *never* (score = 1) or *ever* (scores >1). The psychometric properties of the VOZZ-screen are good (Huisman et al., 2015). In the present study, internal reliability was good (α = 0.81) across the 10 items.

#### Emotional and behavioral problems

The 112-item Youth Self-Report 11–18 (YSR/11–18, (Achenbach & Rescorla, 2001; Verhulst & Van der Ende, 2013)) is widely used to measure emotional and behavioral problems in youth aged 11–18 years old. Adolescents are asked to indicate whether statements were *“Not true”* (0), *“Somewhat or sometimes true”* (1), or *“Very true or often true”* (2) in the past six months. The overarching syndrome scales Internalizing (31 items) and Externalizing problems (32 items) were used. Higher scores indicate more emotional and behavioral problems. The YSR has demonstrated good validity and reliability (Achenbach & Rescorla, 2001), with internal reliability values in the present study being excellent for the internalizing problems scale (α = 0.90) and good for the externalizing problems scale (α = 0.84).

#### Family functioning

The total score of the 12-item General Functioning scale (GF-12) of the McMaster Family Assessment Device (Epstein et al., 1983) was used to measure family functioning. The questionnaire captures respectively healthy and unhealthy aspects of general family functioning, e.g., *“In times of crisis we can turn to each other for support”* and *“We don’t get along well together”*. The parent that accompanied the adolescent to the research center answered all items on a 4-point Likert scale ranging from 1 (*Strongly disagree*) to 4 (*Strongly agree*). In case of two accompanying parents, only one of them filled out this questionnaire. Higher scores on the GF-12 indicate worse levels of family functioning. Previous studies using the GF-12 have reported good psychometric properties (Boterhoven de Haan et al., 2015; Kabacoff et al., 1990), and the internal reliability of the instrument in the current study was good as well (α = 0.87).

#### Social support

Social support was assessed by measuring adolescents’ perceived social support from three sources: family, friends, and a significant other. This assessment was conducted using the 12-item Multidimensional Scale of Social Support (MSPSS, (Zimet et al., 1988)). Each domain consists of four questions on a 3-point Likert scale ranging from 0 (*Never or none*) to 2 (*Often or a lot*). Subscale scores can be calculated by adding all items. Higher scores are indicative of higher levels of perceived social support. The MSPSS has demonstrated good validity and reliability in previous studies (Zimet et al., 1990) and good internal reliability in the present study (respectively Family α = 0.81, Friends α = 0.86, and Significant other α = 0.78).

#### Non-verbal IQ-score

An indication of non-verbal IQ was obtained using two subtests of the Dutch Snijders-Oomen Nonverbal Intelligence Test-Revised for ages 6–40 (SON-R 6–40), which is relatively insensitive to cultural differences (Tellegen & Laros, 2011). Raw test scores on the subsets “Analogies” and “Categories” were combined, multiplied by two and subsequently converted into estimated IQ scores using norms tailored to exact age and sex. IQ scores were adjusted for the Flynn effect, which refers to the rising intelligence test performance in the general population over time and generations (Pietschnig & Voracek, 2015). Previous studies using the SON-R have demonstrated good psychometric properties with strong correlations between the subtests Analogies and Categories and the other subtests and other IQ measures like the WISC-II/IV (Tellegen & Laros, 2011).

#### Self-esteem

The 10-item self-report Rosenberg Self-Esteem Scale (RSES, (Rosenberg, 1965)) measures individuals’ positive and negative attributions about themselves. Items are scored on a 4-point Likert scale, ranging from 0 (*Strongly disagree*) to 3 (*Strongly agree*). The RSES total score used in this study can range from 0–30, with higher scores indicating a higher level of self-esteem. The scale has good predictive validity, as well as internal consistency and test-retest reliability (Gnambs et al., 2018). Internal consistency in the present study was good, α = 0.88.

#### Demographic characteristics

All characteristics were obtained using a demographic questionnaire filled out by the parent or primary caregiver. Ethnic background was coded as either Dutch, or Non-Dutch (e.g., Europe, North America), or non-Western (e.g., Africa, Latin America, Asia including Turkey), based on parental country of birth. Household net monthly income was divided into four categories: ≤1599, 1600–2399, 2400–4399, and ≥4400 euros. Educational level was provided by the adolescent and divided into five categories: special needs, pre-vocational, higher general, pre-university, and mixed secondary educational level (starting high school in a combined-level education).

### Data Analysis

After the identification of adolescents who reported lifetime NSSI, latent class analysis (LCA) was conducted to identify subgroup heterogeneity in self-harming adolescents. LCA was executed using Mplus Version 8.8 (Muthen & Muthen, 2000). Latent class indicators included lifetime NSSI frequency, lifetime number of NSSI methods endorsed, NSSI methods (cutting, biting, burning, carving, pinching, pulling hair, scratching, banging or hitting, rough surfaces), NSSI urgency, pain during NSSI, lifetime suicidal ideation, lifetime suicide attempt (all categorical), and intrapersonal and interpersonal functions of NSSI (both continuous). The method of *Needle sticking* was excluded because of its low prevalence, which resulted in the exclusion of 1 adolescent for LCA analyses.

First, a one-class model was computed to calculate baseline fit indices and one class after another was added to determine the optimal number of latent classes. Model fit was based on parsimony and goodness-of-fit statistics: (1) Bayesian Information Criterion (BIC), (2) Akaike’s Information Criterion (AIC, adjusting for the number of parameters in the model), (3) Sample Size-Adjusted BIC (SSA-BIC), with smaller values of BIC, AIC, and SSA-BIC indicating a better model fit. Models were also compared by examining (4) log-likelihood values, (5) entropy values (preferred to be at least 0.80), the significance of (6) the Lo-Mendell-Rubin Likelihood Ratio Test (LMR-LRT, assesses improvement of fit between competing models), and (7) Bootstrapped Likelihood Ratio tests (BLRT). It was visually examined whether the final model reflected coherent, distinct, and conceptually meaningful subgroups. After the identification of the best-fitting latent class solution, one single categorical variable was created to represent the classes. This variable was used for all follow-up analyses in SPSS IBM Statistics Version 28.0 (IBM Corp., Armonk, NY).

Sociodemographic and clinical differences among the four latent classes of adolescents engaging in NSSI were examined using ANOVAs or Kruskal–Wallis tests for continuous variables and Pearson’s chi-squared tests for categorical variables. Post hoc *z*-tests for independent proportions were conducted as needed (Beasley & Schumacker, 1995).

Subsequently, the class variable was included as the dependent variable in multinomial regression analyses comparing the four latent classes with adolescents who did not report NSSI. Independent variables in the analyses included sex, age, ethnic background, household monthly income, non-verbal IQ score, internalizing and externalizing problems, family functioning, social support from respectively family, friends and significant others, and self-esteem.

Missing data were handled in two ways. For missing items on sum scores of the ISAS-II, YSR, GF-12, MSPSS, and RSES, when at least 75% of the items were valid, the average sum score on valid items was multiplied by the number of items in the scale to estimate scores. Missing values on the YSR, GF-12, MSPSS, and RSES after these calculations and missing values on ethnic background, household monthly income, and non-verbal IQ were handled by multiple imputation. A *p*-value of below 0.05 was considered statistically significant.

## Results

### Sample Characteristics

Table 1 describes baseline sociodemographic and suicidality characteristics for the total sample as well as comparisons between adolescents who reported lifetime NSSI (*n* = 322, 33.3%) versus those who did not (*n* = 644, 66.7%). In the NSSI group, the proportion of girls was significantly larger than the proportion of boys (χ^2^(1) = 18.32, *p* <0.001). In both the NSSI as well as the No-NSSI group, most adolescents had a Dutch ethnic background and followed pre-vocational secondary education. Lifetime suicidal ideation and suicide attempts were more often reported by those who reported lifetime NSSI endorsement (respectively χ^2^(1) = 178.47, *p* < 0.001 and χ^2^(1) = 60.30, *p* < 0.001).

**Table 1:** Sociodemographic characteristics, NSSI, and suicidality status of the study population

	Total sample (*n* = 966)	No-NSSI (*n* = 644)	NSSI (*n* = 322)	Test-statistic	*p*-value
Age—years (*M*, *SD*)	14.9 (0.9)	14.9 (0.8)	15.0 (0.9)	*U* = 11,05787.0	0.091
Age—range	12.7–18.3	12.7–18.3	12.9–18.1		
Sex	*n* (%)	*n* (%)	*n* (%)	χ^2^(1) = 18.3	**<0.001**
Female	500 (51.8)	302 (46.9)	198 (61.5)		
Male	466 (48.2)	342 (53.1)	124 (38.5)		
Ethnic background^a^				χ^2^(1) = 0.02	0.904
Dutch	664 (76.8)	449 (76.9)	215 (76.5)		
Non-Dutch	201 (23.2)	135 (23.1)	66 (23.5)		
Educational level^a^				χ^2^(4) = 1.8	0.760
Special needs secondary education	36 (4.0)	27 (4.4)	9 (3.1)		
Pre-vocational secondary education	409 (45.2)	270 (44.2)	139 (47.4)		
Higher secondary education	204 (22.6)	137 (22.4)	67 (22.9)		
Pre-university education	180 (19.9)	126 (20.6)	54 (18.4)		
Mixed educational level	75 (8.3)	51 (8.3)	24 (8.2)		
Household monthly income^a^				χ^2^(3) = 1.4	
≤1599 euros	103 (12.5)	68 (12.3)	35 (12.9)		0.713
1600–2399 euros	132 (16.0)	87 (15.7)	45 (16.6)		
2400–4399 euros	412 (49.9)	273 (49.3)	139 (51.3)		
>4400 euros	178 (21.6)	126 (22.7)	52 (19.2)		
Lifetime suicidality					
Suicidal ideation	237 (24.5)	74 (11.5)	163 (50.6)	χ^2^(1) = 178.5	**<0.001**
Suicide attempt	40 (4.1)	4 (0.7)	36 (11.2)	χ^2^(1) = 60.3	**<0.001**

*Note*. *M* mean; *SD* standard deviation.

^a^Data regarding ethnic background, educational level, and household monthly income were missing for 101, 62, and 141 adolescents respectively.

Statistical tests conducted were Mann-Whitney *U* tests for continuous data and Pearson’s chi-squared tests for categorical variables. *P*-values that are bolded denote statistical significant findings.

### Extraction of Latent Classes

Table 2 displays the results of latent class analyses for 1-5-class solutions. The 4-class model was the best fit for the data based on the goodness-of-fit indices. No classes contained less than 5% of the total sample. The 4-class model had the lowest BIC value compared to the other classes and a higher entropy value than the 3-class model. Although the 5-class model had lower AIC and SSA-BIC values and a higher entropy value than the 4-class model, the non-significant results of the LMR-LRT test indicated that the 5-class model did not provide a better fit to the data.

**Table 2:** Model fit statistics for latent class analysis specifying 1–5 class solutions

Classes	LL	AIC	BIC	SSA-BIC	Entropy	LMR-LRT, *p*-value	BLRT, *p*-value
1	−5176.77	10401.54	10492.02	10415.93	-	-	-
2	−4873.07	9840.13	10017.39	9868.32	0.846	602.86, <0.001	<0.001
3	−4776.54	9690.98	9954.98	9732.95	0.911	193.70, 0.001	<0.001
**4**	**−4694.77**	**9575.55**	**9926.29**	**9631.31**	**0.933**	**161.29, 0.01**	**<0.001**
5	−4652.67	9537.35	9974.84	9606.90	0.936	83.57, 0.60	<0.001

The bolded row represents the model with the best fit to the data

*LL* Loglikelihood, *AIC* Akaike’s Information Criterion, *BIC* Bayesian Information Criterion, *SSA-BIC* Sample size adjusted BIC, *LMR-LRT* Lo-Mendell-Rubin Adjusted Likelihood Ratio Test, *BLRT* Bootstrapped Likelihood Ratio Test

Class characteristics for the 4-class model are reported in Table 3; conditional probabilities are presented in Appendix 1. Class 1 was the largest (*n* = 108, 33.6%) and was labeled *“Low NSSI–Low suicidality”*. This class was characterized by adolescents who indicated NSSI frequencies mostly in the 1–10 range (90.8%) and none had endorsed more than one method of NSSI. In this group, banging or hitting was by far the most frequently reported method (37.0%), followed by cutting (13.9%) and severe scratching (11.1%). Percentages of lifetime suicidal ideation were comparable to those reported in Class 2, with one adolescent reporting a lifetime suicide attempt. Compared to other classes, adolescents in this class recognized themselves least in statements on motivational background for performing NSSI.

**Table 3:** Characteristics of NSSI and suicidality in the 4-class model

	Class 1 (*n* = 108, 33.6%)	Class 2 (*n* = 95, 29.6%)	Class 3 (*n* = 53, 16.5%)	Class 4 (*n* = 65, 20.2%)
	Low NSSI - Low suicidality	Moderate NSSI- Low suicidality	Moderate NSSI - High suicidality	High NSSI - High suicidality
Suicidal ideation—ever (*n*, %)	30 (27.8)	26 (27.4)	46 (88.5)^a^	60 (92.3)
Suicide attempt—ever (*n*, %)	1 (0.9)	0 (0)	17 (32.1)	18 (27.7)
NSSI Frequency (*n*, %)				
1	38 (35.2)	0 (0)	2 (3.8)	0 (0)
2–10	60 (55.6)	42 (44.2)	21 (39.6)	8 (12.3)
11–50	5 (4.6)	33 (34.7)	22 (41.5)	25 (38.5)
>50	5 (4.6)	20 (21.1)	8 (15.1)	32 (49.2)
NSSI no. of methods (*n*, %)				
1	108 (100)	0 (0)	7 (13.2)	0 (0)
2–3	0 (0)	80 (84.2)	46 (86.8)	0 (0)
>3	0 (0)	15 (15.8)	0 (0)	65 (100)
NSSI Urgency (*n*, %)				
<1 hour	53 (60.9)	61 (67.0)	23 (47.9)	29 (46.0)
1–24 hour(s)	11 (12.6)	12 (13.2)	16 (33.3)	20 (31.7)
>24 hour	23 (26.4)^f^	18 (19.8)^c^	9 (18.8)^d^	14 (22.2)^b^
NSSI Pain (*n*, %)				
No	32 (33.3)	24 (26.4)	11 (21.2)	9 (14.1)
Yes	28 (29.2)	20 (22.0)	17 (32.7)	24 (37.5)
Sometimes	36 (37.5)^e^	47 (51.6)^c^	24 (46.2)^a^	31 (47.7)^a^
Methods (*n*, %):				
Cutting	15 (13.9)	0 (0)	41 (77.4)	47 (72.3)
Biting	8 (7.4)	39 (41.1)	9 (17.0)	29 (44.6)
Burning	1 (0.9)	6 (6.3)	3 (5.7)	19 (29.2)
Carving	11 (10.2)	35 (36.8)	27 (50.9)	53 (81.5)
Pinching	11 (10.2)	50 (52.6)	5 (9.4)	45 (69.2)
Pulling hair	7 (6.5)	24 (25.5)^a^	5 (9.4)	30 (46.2)
Severe scratching	12 (11.1)	30 (31.6)	7 (13.2)	40 (61.5)
Banging or hitting self	40 (37.0)	47 (49.5)	12 (22.6)	50 (76.9)
Rubbing skin against a rough surface	3 (2.8)	18 (19.1)^a^	6 (11.3)	20 (30.8)
NSSI Intrapersonal function (*M, SD*)	19.2 (3.9)	20.1 (3.4)	25.8 (4.3)	26.6 (4.8)
NSSI Interpersonal function (*M, SD*)	27.2 (3.7)	29.8 (6.0)	30.3 (5.4)	31.1 (4.9)

*Note*. ^a^missing data for *n* = 1; ^b^missing data for *n* = 2; ^c^missing data for *n* = 4; ^d^missing data for *n* = 5; ^e^missing data for *n* = 12; ^f^missing data for *n* = 21. *M* mean, *SD* standard deviation

Class 2 was the second largest class (*n* = 95, 29.6%) and was labeled *“Moderate NSSI–Low suicidality”*. This group was composed of adolescents who reported NSSI frequencies predominantly in the 2–50 range (78.9%). They all reported having endorsed more than one NSSI method, but 84.2% did not endorse more than three. The most frequently endorsed methods of NSSI in Class 1 were pinching (52.6%), banging or hitting (49.5%), and biting (41.1%). None of the adolescents in this class stated to have ever cut themselves. Even though 27.4% of adolescents reported lifetime suicidal ideation, none reported a lifetime suicide attempt.

The smallest class in the sample was Class 3 (*n* = 53, 16.5%) and was labeled *“Moderate NSSI–High suicidality”*. The lifetime frequency of NSSI in Class 3 was comparable to that reported by adolescents in Class 2, most of them reported lifetime NSSI frequencies in the 2–50 range (81.1%), but none of the adolescents in Class 3 had endorsed more than three methods. In this group, cutting was reported by a large majority of adolescents (77.4%), followed by carving (50.9%) and banging or hitting (22.6%). Adolescents in this class reported high levels of suicidality; 88.5% reported lifetime suicidal ideation and 32.0% at least one suicide attempt.

The highest levels of both NSSI and suicidality were reported in Class 4 (*n* = 65, 20.2%), which was labeled *“High NSSI–High suicidality”*. Most adolescents reported NSSI frequencies in the 11–50 and >50 ranges (87.7%) and all had endorsed more than three methods of NSSI. A percentage of 81.5% of adolescents in Class 4 reported carving, followed by banging and hitting (76.9%) and cutting (72.3%) as the second and third most frequently endorsed methods. Lifetime suicidal ideation was reported by 92.3% and 27.7% had ever performed a suicide attempt. Compared to other classes, adolescents in this class recognized themselves the most in the statements on the ISAS-II on intrapersonal and interpersonal motivational backgrounds for performing NSSI.

### Class Comparisons

After determining the four latent classes, individuals were assigned to their most likely class. To further explore similarities and differences between classes, they were compared on several demographic and clinical variables. Results are depicted in Table 4. Classes with high levels of suicidality (Classes 3 and 4) comprised a significantly larger proportion of girls and reported significantly higher levels of internalizing problems than classes with low levels of suicidality (Classes 1 and 2). Adolescents in Classes 3 and 4 were more often of a non-Dutch ethnic background than adolescents in Class 1. Reported household income in Class 4 was significantly more often in the 2400–4399 euros range than for adolescents in Class 3 and significantly less often in the highest income level of ≥4400 than in Class 2. Adolescents in Class 4 (“*High NSSI-High suicidality”*) additionally reported more externalizing problems, lower self-esteem, and less social support from their family than adolescents in Classes 1 and 2. Compared to Class 1, Class 4 reported experiencing lower levels of social support from friends as well. Interestingly, adolescents from Class 2 reported the lowest levels of social support from a significant other and herein differed significantly from levels reported by Class 3.

**Table 4:** Sociodemographic and clinical differences among the four latent classes of adolescents engaging in nonsuicidal self-injury

	Class 1 (*n* = 108, 33.6%)	Class 2 (*n* = 95, 29.6%)	Class 3 (*n* = 53, 16.5%)	Class 4 (*n* = 65, 20.2%)	Test statistic	*p*-value	Effect
	Low NSSI - Low suicidality	Moderate NSSI- Low suicidality	Moderate NSSI - High suicidality	High NSSI - High suicidality			
Sex, female (*n*, %)	57 (52.8)	49 (51.6)	42 (79.2)	49 (75.4)	χ^2^(3) = 19.7	**<0.001**	1,2 ≠ 3,4
Age, years (*M*, *SD*)	14.9 (0.9)	14.9 (0.9)	15.2 (1.1)	15.2 (1.0)	*F* = (3,317) = 1.91	0.127	*NS*
Ethnic background (*n*, %)^a^					χ^2^(3) = 14.3	**0.002**	
Dutch	81 (85.3)	64 (75.3)	32 (68.1)	37 (69.8)			1 ≠ 3,4
Non-Dutch	7 (7.4)	15 (17.6)	14 (29.8)	14 (26.4)			1 ≠ 3,4
Household income (*n*, %)^b^					χ^2^(9) = 18.3	**0.032**	
≤1599 euros	10 (11.0)	13 (15.5)	7 (15.2)	4 (8.2)			
1600–2399 euros	15 (16.5)	9 (10.7)	13 (28.3)	8 (16.3)			
2400–4399 euros	47 (51.6)	41 (48.8)	17 (37.0)	34 (69.4)			3 ≠ 4
≥4400 euros	19 (20.9)	21 (25.0)	9 (19.6)	3 (6.1)			2 ≠ 4
Non-verbal IQ score (*Mdn*, *IQR*)	97.0 (19.0)	100.0 (19.0)	97.0 (12.0)	104.0 (19.0)	*H*(3) = 2.40	0.493	*NS*
Internalizing problems (*Mdn*, *IQR*)	14.0 (9.0)	12.0 (10.0)	20.0 (14.0)	21.0 (11.0)	*H*(3) = 60.43	**<0.001**	1,2 < 3,4
Externalizing problems (*Mdn*, *IQR*)	8.0 (8.0)	9.0 (7.0)	12.0 (9.0)	12.0 (9.4)	*H*(3) = 16.81	**<0.001**	1,2 < 4
Family functioning (*Mdn*, *IQR*)	19.0 (8.0)	20.0 (7.0)	22.0 (8.0)	19.0 (8.0)	*H*(3) = 4.83	0.184	*NS*
Social support: Family (*Mdn*, *IQR*)	7.0 (2.0)	7.0 (2.0)	7.0 (4.0)	7.0 (3.0)	*H*(3) = 14.24	**0.003**	1,2 > 4
Social support: Friends (*Mdn*, *IQR*)	7.0 (3.0)	6.0 (2.0)	6.0 (4.0)	5.0 (4.5)	*H*(3) = 11.35	**0.010**	1 > 4
Social support: Significant other (*Mdn*, *IQR*)	8.0 (3.0)	6.0 (3.0)	7.0 (2.0)	6.0 (4.0)	*H*(3) = 13.83	**0.003**	2 < 3
Self-esteem (*Mdn, IQR*)	20.0 (7.0)	19.0 (6.0)	18.0 (12.6)	14.0 (9.5)	*H*(3) = 22.79	**<0.001**	1,2 > 4

Statistical tests conducted were ANOVA’s or Kruskal–Wallis tests for continuous data and Pearson’s chi-squared tests for categorical variables (with Bonferroni correction for multiple tests). *P*-values that are bolded denote statistical significant findings

*IQR* interquartile range, *Mdn* median, *NS* nonsignificant findings

^a^Data on ethnic background was missing for 13, 10, 6, and 12 adolescents

^b^Data on household income was missing for 17, 11, 7, and 16 adolescents

### No NSSI-Group Comparison

To assess differences in clinical constructs between NSSI classes relative to participants who did not report NSSI, multinomial logistic analyses were conducted with adolescents who did not perform NSSI as the reference group. Results are reported in Table 5. Mean item scores and standard deviations for study parameters can be found in Appendix 2. Compared to the No NSSI group, adolescents in Class 3 were disproportionally more likely to be girls. The results state that adolescents in Class 4 were more likely to have a household income in the ≥4400 euros category, but since this category was only applicable for three adolescents there may be a lot of uncertainty in the estimate. It is important to interpret this result with caution, especially since the confidence interval is wide; the true value could potentially be much different from the point estimate. All adolescents that engaged in lifetime NSSI had higher odds of reporting higher levels of internalizing problems than the No NSSI group. Adolescents in Class 4 were additionally more likely to report higher levels of externalizing problems. No differences were found between the reference group and the adolescents reporting NSSI on levels of experienced family support, but adolescents from Classes 2, 3, and 4 had higher odds of rating the experienced support from friends lower than those in the No NSSI group. Interestingly, adolescents from Class 3 were more likely to rate their support from a significant other higher than those who did not self-injure.

**Table 5:** Multinomial logistic regression of No NSSI versus all NSSI classes on sociodemographic characteristics and clinical correlates

		Odds ratio (95% Confidence interval)			
	No NSSI group (*n* = 645)	Class 1 vs No NSSI	Class 2 vs No NSSI	Class 3 vs No NSSI	Class 4 vs No NSSI
Sex (*n*, %)					
Male	342 (53.0)	Ref.	Ref.	Ref.	Ref.
Female	303 (47.0)	1.0 (0.6–1.6)	0.8 (0.5–1.4)	**0.4 (0.2**–**0.9)**^a^	0.6 (0.3–1.3)
Age, years (*M*, *SD*)	14.9 (0.8)	1.0 (0.8–1.3)	0.9 (0.7–1.2)	1.1 (0.8–1.5)	1.2 (0.8–1.7)
Ethnic background (*n*, %)					
Dutch	450 (76.9)	Ref.	Ref.	Ref.	Ref.
Non-Dutch	135 (23.1)	1.7 (0.9–3.1)	1.1 (0.6–1.9)	0.6 (0.3–1.3)	0.7 (0.3–1.5)
Monthly household income (*n*, %)					
≤1599 euros	69 (12.4)	1.2 (0.5–2.6)	1.0 (0.5–2.2)	1.3 (0.5–3.7)	2.9 (0.9–9.6)
1600–2399 euros	87 (15.7)	1.2 (0.6–2.3)	1.8 (0.8–3.9)	0.8 (0.3–1.8)	2.4 (0.8–7.3)
2400–4399 euros	273 (49.2)	Ref.	Ref.	Ref.	Ref.
≥4400 euros	126 (27.7)	1.1 (0.6–1.9)	0.9 (0.5–1.7)	0.9 (0.4–2.4)	**4.9 (1.6**–**15.8)**^b^
Non-verbal IQ score (*Mdn*, *IQR*)	97.0 (6.0)	1.0 (1.0–1.0)	1.0 (1.0–1.0)	1.0 (1.0–1.0)	1.0 (1.0–1.0)
Internalizing problems (*Mdn*, *IQR*)	9.0 (9.0)	**1.1 (1.0**–**1.1)**^b^	**1.1 (1.0**–**1.1)**^b^	**1.1 (1.1**–**1.2)**^c^	**1.1 (1.1**–**1.2)**^c^
Externalizing problems (*Mdn*, *IQR*)	8.0 (8.0)	1.0 (1.0–1.1)	1.0 (1.0–1.1)	1.0 (1.0–1.1)	**1.1 (1.0**–**1.1)**^b^
Family functioning (*Mdn*, *IQR*)	19.0 (7.0)	1.0 (1.0–1.0)	1.0 (1.0–1.1)	1.0 (1.0–1.1)	1.0 (0.9–1.1)
Social support: Family (*Mdn*, *IQR*)	8.0 (1.0)	1.0 (0.8–1.1)	1.0 (0.9–1.2)	1.0 (0.8–1.2)	0.9 (0.7–1.0)
Social support: Friends (*Mdn*, *IQR*)	8.0 (2.0)	0.9 (0.8–1.0)	**0.8 (0.7**–**1.0)**^a^	**0.8 (0.7**–**1.0)**^a^	**0.8 (0.7**–**0.9)**^b^
Social support: Significant other (*Mdn*, *IQR*)	7.0 (3.0)	1.0 (0.9–1.2)	0.9 (0.8–1.1)	**1.2 (1.0**–**1.5)**^a^	1.0 (0.9–1.2)
Self-esteem (*Mdn*, *IQR*)	21.0 (6.0)	1.0 (0.9–1.1)	1.0 (0.9–1.0)	1.0 (0.9–1.1)	0.9 (0.9–1.0)

*R*^*2*^ = 0.32 (Cox-Snell), 0.36 (Nagelkerke). Model χ^2^(56) = 199.00, *p* < 0.001. Bolded data represent significant findings

*Mdn* median, *IQR* interquartile range, *Ref* reference group

^a^*p* < 0.05, ^b^*p* < 0.01, ^c^*p* < 0.001

## Discussion

NSSI is frequently encountered in adolescents, but its predictive value for suicidality or other clinical characteristics is difficult to determine because of its heterogeneous nature. Despite the frequent co-occurrence of NSSI and suicidality, it is noteworthy that they have not often been considered together as potential predictors in studies examining their relationship with other variables and if so, studies focused on late adolescents, student populations, adults or clinical samples. This study aimed to identify distinct subgroups of adolescents based on their history of NSSI and suicidality in a large population-based cohort of adolescents oversampled for their risk of emotional and behavioral problems. By comprehensively investigating the relationship between NSSI and suicidality in adolescents, with an additional focus on understanding the underlying risk factors and psychosocial correlates, the study intended to provide tools for targeted prevention and intervention strategies. This could ultimately enhance our ability to support the mental well-being of adolescents.

Consistent with previous research, girls reported significantly more often to have engaged in lifetime NSSI than boys (Bresin & Schoenleber, 2015). Lifetime suicidal ideation and suicide attempts were more often reported by those who reported lifetime NSSI endorsement. The percentage of adolescents reporting NSSI in our sample (33.3%) was higher than those reported in previous studies but can be explained by the oversampling method used in constructing the cohort. Four mutually exclusive classes of adolescents with lifetime engagement in NSSI were identified by means of latent class analysis. Classes differed on the number of methods used, frequency of NSSI, type of NSSI method, the motivational background of the behavior and levels of suicidality. In follow-up analyses between subgroups of adolescents performing NSSI, distinctions between classes with low levels of suicidality versus those with high levels of suicidality were notable when examining clinical differences between the four classes.

Both classes with high levels of suicidality (Classes 3 and 4) were comprised of a significantly larger proportion of girls than Classes 1 and 2. When compared to adolescents that did not report NSSI, adolescents in Class 3 (“Moderate NSSI-High Suicidality”) were disproportionally more like to be girls. These findings were as expected, since female adolescents are generally more likely to report suicidal ideation compared to males. The overrepresentation of females in the high suicidality classes might also explain the high percentages of cutting and carving reported by adolescents in Class 3 and 4, since previous research already described these behaviors as most often reported by girls (Barrocas et al., 2012).

Although in general no differences on ethnic background were found when comparing adolescents that reported lifetime NSSI to those that did not, the high suicidality classes did include a higher percentage of adolescents with a non-Dutch background than Class 1 (“Low NSSI-Low Suicidality”), in which over 85 percent of adolescents had a Dutch ethnic background. Higher levels of NSSI, suicidal ideation and suicide attempts have been found in previous studies in adolescents with a migration background (Donath et al., 2019), yet, the findings from the present study should be interpreted with caution. The cultural diversity within non-Dutch populations means that experiences with NSSI can vary widely depending on specific cultural, regional, and individual factors. Future research in this area should continue to explore and respect these nuances.

Consistent with findings previous studies employing LCA (Case et al. (2020); Dhingra et al., 2015; Hamza & Willoughby, 2013; Klonsky & Olino, 2008; Marraccini et al. (2021)), adolescents reporting high levels of suicidality (Classes 3 and 4) reported higher levels of internalizing problems than adolescents with low levels of suicidality (Classes 1 and 2). This difference is of particular interest when considering that both Classes 2 and 3 report moderate levels of NSSI. The higher levels of internalizing problems might trigger higher levels of suicidality for those adolescents in Class 3. Overall, all adolescents reporting NSSI engagement had higher odds of reporting internalizing problems than those that did not engage in NSSI. Class 4, with high levels of both NSSI and suicidality, additionally reported higher levels of externalizing problems than other self-injuring adolescents and increases in the level of externalizing problems increased the odds of being in this class instead of in the No NSSI group. This is in line with reported findings on associations between NSSI and externalizing behaviors in a systematic review that included ADHD, conduct disorders, antisocial personality disorders and oppositional defiant disorders as risk factors for engagement in NSSI. The authors stated that it might be interesting to further investigate the hypothesis that externalizing problems might be more strongly associated with NSSI, while internalizing problems might play a bigger role in suicidality (Meszaros et al., 2017). Adolescents in Class 4 reported experiencing lower levels of self-esteem as well compared to other adolescents endorsing NSSI. Comparable results have been found in first-year students (Hamza & Willoughby, 2013).

Family functioning and support have previously been named as protective factors for NSSI in adolescence (Wang et al., 2022). In the present study, it was found that adolescents with high levels of both NSSI and suicidality, reported less experienced social support from their family than adolescents with low levels of suicidality. A supportive family environment provides emotional validation, coping resources, and a sense of belonging, which can help individuals better navigate difficult emotions and challenges. Yet, the shift from relying primarily on family to seeking support and companionship from friends is a common and natural developmental transition during adolescence (Rosenthal & Kobak, 2010). The current results might underline this shift: adolescents in Classes 2–4 (from moderate to high NSSI) were at higher odds to report lower levels of social support by friends than those in the low NSSI class and adolescents that did not report lifetime NSSI engagement. It could be that adolescents who experience higher levels of social support from friends at this age, feel more competent in handling daily life stressors. It was however interesting that adolescents in Class 3, “Moderate NSSI-High Suicidality”, were disproportionally more likely to report the highest levels of social support from a significant other. To be able to explain this finding, it might be important to know more about the relationship of the adolescent with the significant other. Is the relationship a supportive one and does it help to reduce the likelihood of engaging in even more severe NSSI? Or is the significant other unsupportive, abusive, or causing significant stress and contributes this to an increased risk of NSSI? (Levesque et al., 2010) If the significant other engages in self-harming behaviors, it may normalize or validate NSSI for the individual, which can potentially lead to an increase in self-harming behaviors or hinder efforts to seek healthier coping strategies (Claes, Houben et al., 2010).

The current study design enabled comparison of classes of self-injuring individuals amongst themselves and with adolescents who did not report NSSI. Results of these analyses revealed that especially the experience of higher levels of internalizing problems and lower levels of social support by friends compared to those in the No NSSI group might be a topic of interest for future research. This diminished ability to discuss emotional challenges with peers might propel some adolescents towards NSSI as a coping strategy. Findings on ethnic background for the high suicidality classes and the support of significant others for some adolescents performing NSSI are in need of further exploration as well.

Overall, adolescents in the low suicidality classes might be least at risk for adverse clinical outcomes than those who did report suicidality. Class 4, “High NSSI-High Suicidality”, is especially concerning, since adolescents in this class faced the highest increased odds of adverse clinical outcomes compared to the No NSSI group, as well as to other adolescents that reported NSSI. This emphasizes the urgency of multidimensional interventions tailored for this particular group. Considering the age of adolescents in the present sample, which coincides with the typical onset of NSSI (Plener et al., 2015), it could be that adolescents in the classes with low suicidality have mostly been experimenting with NSSI, whereas for adolescents in classes with high levels of suicidality, NSSI already consequently serves as a coping mechanism for dealing with emotional frustration or pain. Motivational background could be an important factor in maintenance or cessation of NSSI; a combination of interpersonal and intrapersonal motivational background contributes to the onset and maintenance of NSSI in adolescence (Tatnell et al., 2014). Accordingly, the results show that adolescents from the class with the highest levels of NSSI and suicidality recognized themselves most in both functions of NSSI compared to other classes.

Consistent with previous studies (Whitlock et al., 2008), it was found that the co-occurrence of suicidality and NSSI was associated with greater psychological and psychosocial impairments compared to NSSI alone. This finding underlines the complexity of NSSI, supporting the argument for the necessity of examining these behaviors across a continuum, rather than strictly separating them as distinct constructs (Dhingra et al., 2016). The Experiental Avoidance model describes the primary function of deliberate self-harm as “the avoidance of, or escape from, unwanted or aversive states of emotional arousal” (Chapman et al., 2006). Engaging in deliberate self-harm can lead to temporary relief and over time, this way deliberate self-harm can become a more automatic, conditioned response to emotional arousal. The current finding that more severe NSSI was associated with higher levels of suicidality may additionally support Joiner’s Interpersonal Theory of Suicide (Joiner, 2005). This theory states that in general, people avoid pain and fear death. In the present study too, most adolescents did not report lifetime NSSI or suicidality. To be able to end one’s life, an individual needs to have both suicidal ideation and the capability to act on this desire. By performing NSSI as a form of temporary relief as stated in the Experiental Avoidance model, one might overcome this fear of pain and increase the capability for the performance of suicidal behavior. When frequently endorsing NSSI, this behavior might lose its function of temporary relief and increase the chances of transitioning to the aforementioned suicidal behavior (Klonsky et al., 2013).

This study benefits from several strengths, including a large high-risk sample in which one third of the adolescents reported lifetime NSSI endorsement. Another strength is the focus on middle adolescence, a critical developmental period associated with the onset of many psychopathological issues. Since the majority of NSSI behaviors typically initiate at the age of 12 and 14 years (Cipriano et al., 2017), with the highest prevalence reported in middle adolescence (Brown & Plener, 2017) and a decrease towards late adolescence (Plener et al., 2015), the present study sample with a mean age of 14.9 years old potentially captures the inception of these behaviors. To our knowledge, this study is the first study to have used latent class analysis to identify subgroups of adolescents based on NSSI and suicidality in a sample this young of age. Moreover, using the ISAS instrument allowed for a more in-depth and varied assessment of nonsuicidal self-injuring behaviors, while using the VOZZ-screen enabled us to study suicidal ideation as well as suicide attempts. Additionally, the design of the study allowed us to examine associations between self-harm and a broad range of sociodemographic characteristics and clinical outcomes, as well as compare adolescents who perform NSSI to those who do not.

This study has some limitations as well. The cross-sectional design prevents determining the directionality of relationships between lifetime NSSI and suicidality and associated clinical outcomes. Future studies, including follow-up measurements in the present cohort, can benefit from longitudinal designs to offset this limitation. Considering the sensitive nature of self-harming behavior and the persistent stigma surrounding the topic, there is a possibility of underreporting. However, we maintain that adolescents are, despite these challenges, the most reliable sources for disclosing information regarding this issue. When adolescents visited the research center, researchers emphasized that all data would be processed anonymously. In case of underreport in the present study, one could argue that this would predominantly have led to misclassification in the groups reporting no or lower levels of NSSI. Redistribution of these adolescents to groups with higher levels of NSSI, would make already found associations even stronger. Another limitation could be the possibility of recall bias in remembering the lifetime frequency of NSSI and suicidal ideation. Especially when an adolescent reported to have endorsed multiple methods of NSSI with a higher frequency, estimations might have deviated from the actual lifetime NSSI frequency. This issue was addressed in statistical analyses by grouping all reported frequencies above 50 in one category.

The present results not only illuminate crucial areas for future exploration but also underscore the influence of societal and cultural factors on self-harming behaviors. While the four latent classes identified bear resemblance to those from prior LCA studies, their proportions and characteristics diverge, largely due to sociodemographic variances. This accentuates the need for cross-cultural studies to deeply comprehend these discrepancies and the role of environment and culture in shaping self-harming tendencies. Investigating the developmental trajectories of NSSI classes and their long-term impact on adult mental health is pivotal. Additionally, understanding the interplay of potential moderators, such as social support, and mediators like coping strategies, is essential for crafting timely interventions. With a noted higher prevalence of NSSI among girls, probing into sex-specific influences is imperative. One should, however, always be aware of the higher risk for male adolescents on suicide death (Miranda-Mendizabal et al., 2019). These research avenues are integral for enhancing clinical assessment, treatment approaches, and support mechanisms for adolescents grappling with NSSI.

## Conclusion

Nonsuicidal self-injury (NSSI) is frequently encountered in adolescents, but its predictive value for suicidality or other clinical characteristics is complicated by its heterogeneous nature. This research contributes significantly to our understanding of this heterogeneity within the self-injury population, while simultaneously enabling comparisons with prior findings on LCA in self-injury. The findings indicate that adolescents classified into different NSSI classes exhibit variations in reported social support, self-esteem, internalizing and externalizing problems, as well as demographic characteristics. The dynamic interplay between NSSI, suicidality, and associated problems underscores the importance of assessments and interventions that address these issues simultaneously. For clinical practice, the findings emphasize that addressing internalizing problems remains a critical component of any comprehensive approach to preventing and treating self-harm. Evidence-based treatments for internalizing problems, such as psychotherapy and medication, can be effective in reducing self-harm risk. For adolescents presenting with high levels of both NSSI and suicidality, it is recommended to incorporate an in-depth exploration of externalizing problems and assess available social support from family members, Efforts to enhance the adolescent’s self-esteem should also be integrated into the treatment plan. The quality of peer relationships can exert a significant impact on an adolescent’s mental well-being, underscoring the paramount importance of exploring and nurturing these connections for adolescents presenting with NSSI. In conclusion, this study’s results offer insights into NSSI among adolescents and underline the importance of adapting a comprehensive, personalized, multi-faceted, and longitudinal approach in future research and clinical practice.

## Data Availability

This manuscript’s data will not be deposited.

## Appendix

Tables 6, 7

**Table 6:** Conditional probabilities of item endorsement in the four-class model

	Class 1 (*n* = 108, 33.6%)	Class 2 (*n* = 95, 29.6%)	Class 3 (*n* = 53, 16.5%)	Class 4 (*n* = 65, 20.2%)
Suicidal ideation—ever	0.271	0.270	0.867	0.919
Suicide attempt—ever	0.014	0.000	0.287	0.280
NSSI Frequency				
1	0.359	0.000	0.033	0.000
2–10	0.548	0.434	0.428	0.117
11–50	0.047	0.345	0.395	0.393
>50	0.046	0.218	0.144	0.490
NSSI no. of methods:				
1	1.000	0.000	0.154	0.000
2–3	0.000	0.831	0.846	0.000
>3	0.000	0.169	0.000	1.000
NSSI Urgency:				
<1 hour	0.618	0.660	0.490	0.461
1–24 h(s)	0.115	0.831	0.325	0.325
>24 h	0.267	0.169	0.185	0.213
NSSI Pain				
No	0.345	0.275	0.188	0.131
Yes	0.282	0.220	0.348	0.369
Sometimes	0.373	0.505	0.185	0.500
Methods				
Cutting	0.137	0.000	0.721	0.731
Biting	0.076	0.426	0.164	0.435
Burning	0.009	0.060	0.060	0.296
Carving	0.099	0.367	0.495	0.823
Pinching	0.104	0.542	0.095	0.686
Pulling hair	0.063	0.263	0.106	0.451
Severe scratching	0.113	0.325	0.123	0.619
Banging or hitting self	0.375	0.494	0.241	0.768
Rubbing skin against a rough surface	0.026	0.173	0.141	0.315

**Table 7:** Mean item scores and standard deviations for study parameters

	No NSSI Group (*n* = 645)	Class 1 (*n* = 108)	Class 2 (*n* = 95)	Class 3 (*n* = 53)	Class 4 (*n* = 65)
		Low NSSI - Low suicidality	Moderate NSSI- Low suicidality	Moderate NSSI - High suicidality	High NSSI - High suicidality
Non-verbal IQ score (*M, SD*)	100.5 (14.0)	98.9 (11.5)	100.7 (13.9)	97.5 (13.7)	100.7 (14.0)
Internalizing problems (*M, SD*)	9.8 (6.8)	14.1 (7.4)	13.2 (8.0)	20.4 (9.1)	24.0 (10.1)
Externalizing problems (*M, SD*)	8.2 (5.3)	9.7 (5.6)	9.8 (6.1)	12.7 (7.2)	13.7 (7.8)
Family functioning (*M, SD*)	19.3 (4.6)	19.2 (4.9)	20.4 (5.0)	20.6 (5.5)	19.2 (4.8)
Social support: Family (*M, SD*)	7.1 (1.5)	6.5 (1.7)	6.7 (1.4)	6.0 (2.1)	5.8 (2.5)
Social support: Friends (*M, SD*)	6.7 (1.8)	6.3 (2.0)	5.6 (1.8)	5.6 (2.6)	5.0 (2.5)
Social support: Significant other (*M, SD*)	6.0 (2.2)	6.0 (2.3)	5.2 (2.3)	6.8 (1.6)	6.1 (1.8)
Self-esteem (*M, SD*)	20.3 (4.8)	19.5 (5.5)	19.3 (4.6)	16.4 (6.8)	14.0 (6.1)

*M* mean, *SD* standard deviation
